# Identification of different types of tumors based on photoacoustic spectral analysis: preclinical feasibility studies on skin tumors

**DOI:** 10.1117/1.JBO.28.6.065004

**Published:** 2023-06-13

**Authors:** Mengjiao Zhang, Long Wen, Chu Zhou, Jing Pan, Shiying Wu, Peiru Wang, Haonan Zhang, Panpan Chen, Qi Chen, Xiuli Wang, Qian Cheng

**Affiliations:** a Tongji University, Institute of Acoustics, School of Physics Science and Engineering, Shanghai, China; b Tongji University, Institute of Photomedicine, Shanghai Skin Disease Hospital, School of Medicine, Shanghai, China; c National Key Laboratory of Autonomous Intelligent Unmanned Systems, Shanghai, China; d Frontiers Science Center for Intelligent Autonomous Systems, Ministry of Education, China

**Keywords:** photoacoustic spectral analysis, tumor-related biomarkers, endogenous chromophores, support vector machine

## Abstract

**Significance:**

Collagen and lipid are important components of tumor microenvironments (TME) and participates in tumor development and invasion. It has been reported that collagen and lipid can be used as a hallmark to diagnosis and differentiate tumors.

**Aim:**

We aim to introduce photoacoustic spectral analysis (PASA) method that can provide both the content and structure distribution of endogenous chromophores in biological tissues to characterize the tumor-related features for identifying different types of tumors.

**Approach:**

*Ex vivo* human tissues with suspected squamous cell carcinoma (SCC), suspected basal cell carcinoma (BCC), and normal tissue were used in this study. The relative lipid and collagen contents in the TME were assessed based on the PASA parameters and compared with histology. Support vector machine (SVM), one of the simplest machine learning tools, was applied for automatic skin cancer type detection.

**Results:**

The PASA results showed that the lipid and collagen levels of the tumors were significantly lower than those of the normal tissue, and there was a statistical difference between SCC and BCC (p<0.05), consistent with the histopathological results. The SVM-based categorization achieved diagnostic accuracies of 91.7% (normal), 93.3% (SCC), and 91.7% (BCC).

**Conclusions:**

We verified the potential use of collagen and lipid in the TME as biomarkers of tumor diversity and achieved accurate tumor classification based on the collagen and lipid content using PASA. The proposed method provides a new way to diagnose tumors.

## 1 Introduction

In recent years, an increasing number of studies have shown that tumors are closely related to tumor microenvironments (TMEs).^1^^–^^3^ The TME is a complex system mainly composed of blood vessels, collagen, lipid, etc.^3^^–^^5^ Lipid can provide energy for tumors and collagen mainly constitutes the scaffold of the tumor.^6^^,^^7^ Collagen and lipid content can affect the proliferation and differentiation of tumor cells.^8^^–^^11^ Alterations in the content of lipid and collagen are hallmarks of many diseases, including breast cancer,^12^ prostate cancer,^13^^,^^14^ renal cell carcinoma,^15^ skin cancers,^16^ and others. Therefore, the collagen and lipid content in the TME can be used for diagnosis and differentiation of tumors.

Currently, several methods are available for detecting the lipid and collagen of tumors. Second-harmonic generation microscopy, which relies on the nonlinear interaction of a laser with non-centrosymmetric molecules, has been used to image fibrillar collagen within tissues.^17^ It can provide submicron resolution but has limited detection depth and does not provide information about lipid. Magnetic resonance imaging using chemoselective fat-suppression pulse sequences enables lipid detection. However, it fails to provide effective contrast when there is less fat within the tissue.^18^ In recent years, Raman spectroscopy has been successfully used to determine the molecular composition and structure based on the inelastic scattering of different molecules to light.^19^ However, the penetration depth of Raman spectroscopy is limited, and only superficial skin information can be obtained.^19^^,^^20^ To overcome these issues, new diagnostic methods with high sensitivity that can detect both collagen and lipid with sufficient penetration depths are required.

Photoacoustics (PA) is a novel non-invasive detection technique that combines high optical absorption contrast with the high penetration depth of ultrasound.^21^ PA is a physical process of “light in and sound out.”^22^ A pulsed laser is used to irradiate biological tissues wherein the energy is wavelength-selectively absorbed by endogenous chromophores within the tissues, generating ultrasonic waves (PA signals) through thermoelastic expansion. Because ultrasonic waves scatter much less than optical waves, PA technology has a greater detection depth than optical detection technology, which shows great promise for clinical applications.^23^^,^^24^ The endogenous chromophores, such as hemoglobin, collagen, lipid, and water, have different optical absorption spectra in the visible and infrared bands.^25^ By irradiating biological tissues with pulsed lasers of different wavelengths, PA can provide rich endogenous chromophores information about tumors.^26^^–^^29^ Lei et al.^30^ investigated the feasibility of assessing collagen contents to detect fibrosis in Crohn’s disease using PA imaging. Wilson et al.^31^ implemented multiparametric spectroscopic PA imaging to assess the lipid content, oxygen saturation, and total hemoglobin to identify the development of four types of breast cancer. Conventional PA imaging is mainly based on the amplitude of the envelope of time-domain PA signals to quantify the endogenous chromophore concentrations in biological tissues,^32^^,^^33^ ignoring the frequency and phase information of PA signals associated with the absorbers. Besides, the envelope of the time-domain PA signal is subjected to the effects of noise and transducer response. It is quite difficult to achieve reliable results in quantifying the size and concentration of absorber with sizes smaller than the system resolution. Considering the different sizes of the absorbers, the ultrasonic spectrum shows significant advantages. PA spectral analysis (PASA) method can remove the low-frequency system noise and high-frequency measurement noise to provide objective results and repeatable measurements.^34^^,^^35^ Further, frequency analysis has proved feasible in detecting absorbers with sizes smaller than the system resolution.^36^^,^^37^ Recently, PASA, which analyzes the frequency domain power distribution of PA signals, has demonstrated the ability to assess the content and corresponding microstructure of endogenous chromophore in biological tissues simultaneously.^38^^–^^41^ Moreover, the spectral parameters extracted from the PA spectrum, e.g., slope, power-weighted mean frequency, can be used to characterize the microstructures of endogenous optical absorbers.^38^^,^^42^^–^^44^ Xu et al.^39^ implemented the PASA to assess the changes of lipid for fatty liver identification. They further quantified the Gleason score of prostate cancer based on the tissue microscopic architecture using PASA.^45^ Our group combined the PASA with machine leaning to better mine the data information and achieved a high diagnostic accuracy of prostate cancer,^46^^–^^49^ osteoporosis,^50^ and breast cancer.^51^^,^^52^ PASA has shown considerable potential in evaluating the endogenous chromophore in biological tissues for tumor diagnosis.

Therefore, in this study, we investigated the feasibility of non-invasive PASA for characterizing the tumor-related features of lipid and collagen content in the TME to identify different types of tumors. We took skin cancers as the research objects, for which an invasive biopsy is the gold standard for diagnosis. *Ex vivo* experiments were conducted using three types of human skin tissue: normal, squamous cell carcinoma (SCC), and basal cell carcinoma (BCC). The content of lipid and collagen in skin tissue was calculated semi-quantitatively by PASA at different wavelengths. With the help of machine learning classification methods, tumors were successfully identified and tumor types were automatically classified based on quantified PASA parameters.

## 2 Materials and Methods

### 2.1 Ethics Statement

The study protocol was approved by the Ethical Committee of the Shanghai Skin Disease Hospital and was performed in accordance with the tenets of the Declaration of Helsinki. All patients were informed of the purpose of the study, and written consent was obtained before recruitment and sampling.

### 2.2 Sample Collection

A total of 39 patients were enrolled in this study, including 15 with suspected SCC and 12 with suspected BCC. Normal samples were collected from the skin collection areas of 12 patients who received skin grafts. All samples were procured from the Institute of Photomedicine, Shanghai Skin Disease Hospital, School of Medicine, Tongji University, China. After surgical excision of the skin tissue, the residual blood on the tissue surface was cleaned with sterile gauze. Each sample had a diameter of ∼5  mm. The skin tissues were placed in sterile sample tubes, stored in a portable medical cryostat (2°C to 8°C), and transported to the Institute of Acoustics of Tongji University laboratory for testing within 1 h.

### 2.3 Experiment Setup and PA Measurements

**Fig. 1:**
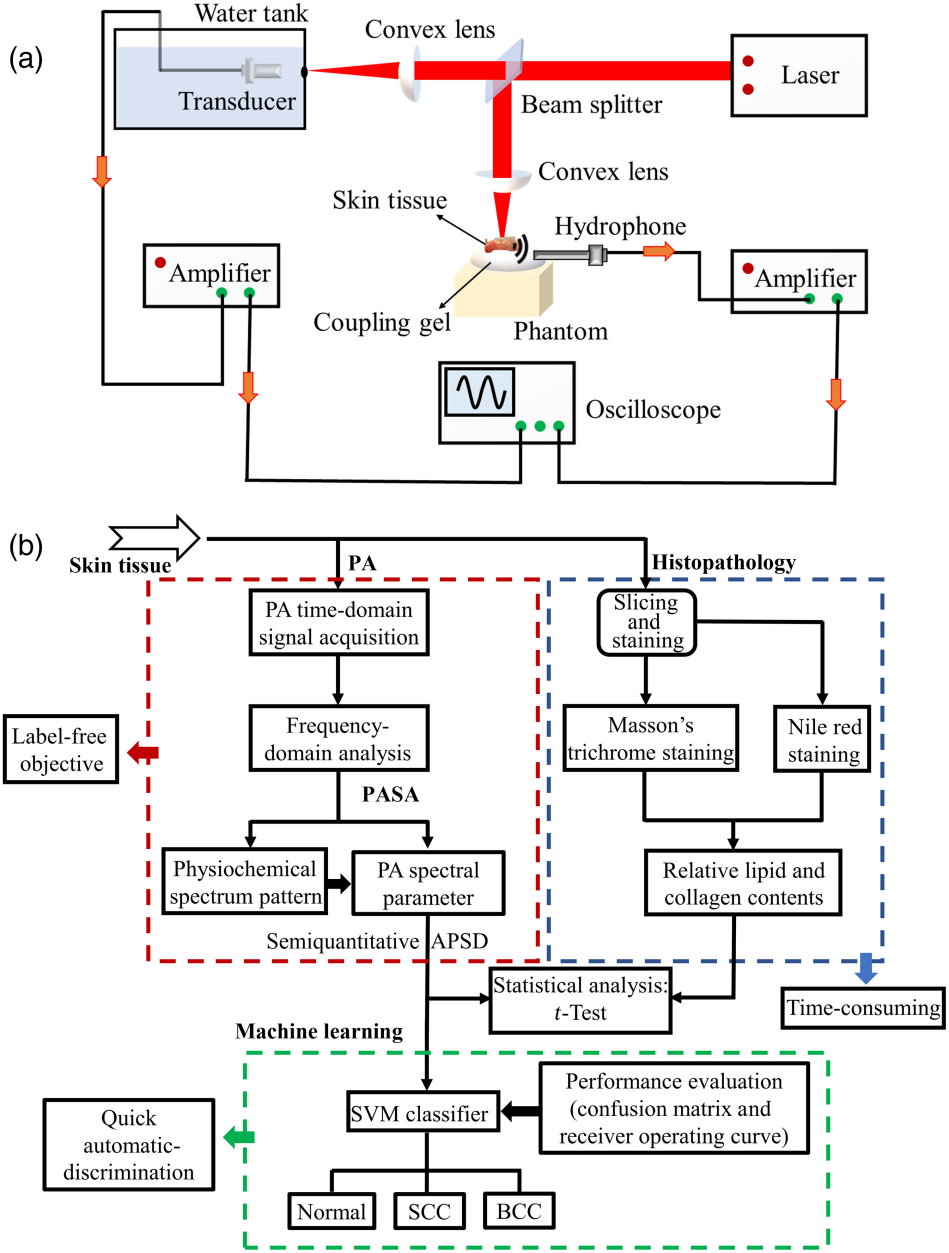
PA experimental system and experiment detection method. (a) Experimental setup for performing *ex vivo* PA measurements. (b) Block diagram of the experimental method. The red, blue, and green dotted boxes represent PA experiments and data processing, histopathology analysis, and machine learning, respectively.

Figure 1(a) shows a schematic of the PA experimental setup. An optical parametric oscillator system pumped by a Nd:YAG laser (Phocus Mobile, OPOTEK, Carlsbad, California, United States) was used to provide laser pulses with wavelengths ranging from 1200 to 1700 nm in 10 nm intervals, covering the strong absorption ranges of lipid and collagen. The laser energy output over the entire wavelength range was controlled to 0.1 to 0.5 mJ per pulse, with a pulse duration of 5 ns and pulse repetition rate of 10 Hz. A laser beam with a diameter of 3 mm illuminated the skin tissue, leading to an optical energy density of 7 to $14\;\mathrm{mJ}/{\mathrm{cm}}^{2}$, which was below the safety limit specified by the American National Standards Institute. As shown in Fig. 1(a), skin tissue was placed on the phantom to avoid strong scattering of the sound signal by any hard boundary. The PA signals generated by the entire laser irradiation of the skin tissues were received by a needle hydrophone with a bandwidth of 1 to 20 MHz (HNC1500, ONDA Corp., Sunnyvale, California, United States). The laser energy varies with the wavelength and fluctuates only slightly over time. To determine the laser energy variations during the PA measurements, 10% of the laser energy was projected onto a black body, and the PA signals generated by the black body were received by a 5 MHz focused transducer (V326, Immersion Transducers, Olympus Corp., Tokyo, Japan). After amplification (5072PR, Olympus Corp., Tokyo, Japan) to boost the PA signals of the skin tissue samples by 25 dB, they were averaged 64 times and recorded using a digital oscilloscope (HDO6000, Teledyne Lecroy, New York, United States) at a sampling rate of 2500 MHz. To improve the stability and reduce the measurement error, PA signals from each skin tissue sample were detected at two different positions. We developed an efficient automated experimental program that includes laser wavelength switching, triggering, and data acquisition. With this program, PA data acquisition of each skin tissue sample and the blackbody could be completed in <13  min, covering 51 wavelengths (1200 to 1700 nm), with an average of 64 times per wavelength.

### 2.4 PA Spectral Analysis

**Fig. 2:**
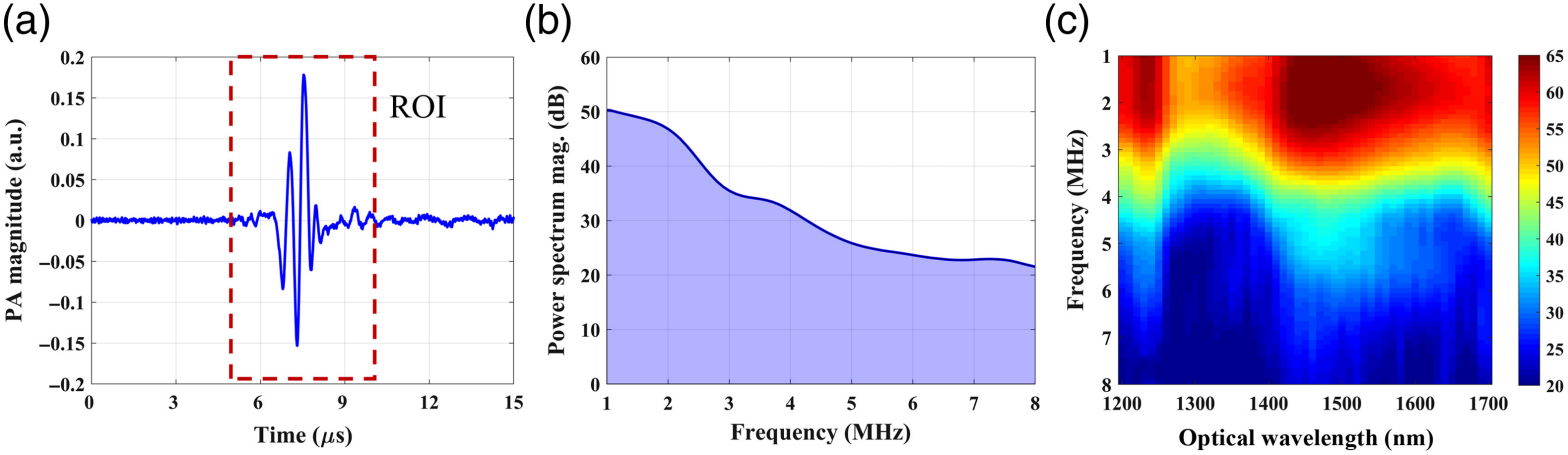
(a) Representative PA signal from skin tissue. The red dotted box indicates the region of interest. (b) Example of the power spectrum density of a PA signal generated by skin tissue. The purple area indicates the APSD. (c) Example of a PAPCS for skin tissue.

The PA signals were analyzed using MATLAB software (R2019b, MathWorks, Natick, Massachusetts, United States). First, the PA signal generated by the skin tissue was calibrated using the peak-to-peak value of the PA signal generated by the black body at each wavelength. Second, based on the calibrated PA signals for each skin tissue sample, the power spectra of the PA signals [Fig. 2(a)] were calculated using Welch’s method with a 5  μs moving Hamming window and 60% overlap, as shown in Fig. 2(b). Considering the signal-to-noise ratio, the ultrasound frequency was first analyzed in the 1 to 8 MHz range. The power spectra of the PA signals obtained in the 1200 to 1700 nm wavelength range were combined to form a PA physiochemical spectrogram (PAPCS), as depicted in Fig. 2(c). The horizontal axis of the PAPCS is the optical wavelength, representing the relative optical absorption of different endogenous chromophores in skin tissue, whereas the vertical axis is the ultrasonic frequency, revealing the structural distribution corresponding to different optical absorptions in skin tissue. The color bar represents the amplitude of the power spectrum. Differences in the lipid and collagen content and in the microstructure of the TMEs of different tumors will form unique PAPCSs.

Furthermore, the changes of relative lipid and collagen content in skin tissues were quantitatively analyzed. Based on the PA power spectral analysis at a wavelength of λ nm [Fig. 2(b)], we calculated the relative area of power spectrum density (APSD) at the corresponding wavelength as follows:

$$\text{Relative }{\mathrm{APSD}}_{\lambda }=\frac{{\left(\int_{f_{0}}^{f_{1}}p(f)df\right)}_{\lambda }}{{\left(\int_{f_{0}}^{f_{1}}p(f)df\right)}_{\lambda_{0}}},$$ (1)

where p(f) is the power spectral density at each frequency, $f_{0}=1\;\mathrm{MHz}$, and $f_{1}=8\;\mathrm{MHz}$. The PA signal is affected by low-frequency system noise and high-frequency measurement noise. Based on the PA power spectrum analysis, setting a high cut-off frequency $f_{1}$ can help avoid high-frequency noise, whereas setting a low cut-off frequency $f_{0}$ can help minimize system noise. Considering the signal-to-noise ratio and the cluster size of lipid and collagen obtained from histology images (see Fig. 4), we set the low and high cutoff frequencies to 1 and 8 MHz, respectively. The PA power spectral density in the specified frequency range was then summarized as the PA absorption value at each wavelength. A reference wavelength $\lambda_{0}$ (690 nm) was used to eliminate systematic errors. We then obtained the relative PA absorption of each skin tissue sample with reduced system noise. To improve the stability and reduce the measurement error, PA signals from each skin tissue sample were detected at two different positions. The relative APSD values from two different directions were then obtained and averaged for further analysis. The relative APSD obtained at wavelength λ (nm) reflects the relative optical absorption of the corresponding endogenous chromophore; thus, it is related to the relative endogenous chromophore content of the skin tissue. According to the literature, lipid exhibits strong absorption at ∼1200 to 1240 nm.^25^^,^^53^^,^^54^ Some studies have also shown that at 1600 to 1700 nm excitation,^55^^–^^57^ the lipid PA signal is enhanced compared to that at 1200 nm excitation. In addition, the selective detection of collagen at ∼1300 to 1340 nm can be achieved.^46^^,^^58^ Thus, to reduce the measurement error caused by a single wavelength, the relative APSD obtained at the absorption wavelength range of lipid (or collagen) were averaged. To examine whether the changes in collagen and lipid content of TME in different types of skin tissues were statistically significant, unpaired t-tests were performed using GraphPad Prism 9.0.

### 2.5 Support Vector Machine Analysis

**Fig. 3:**
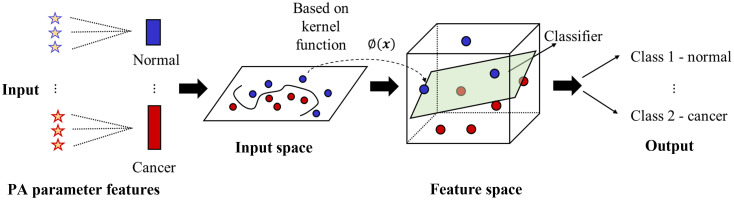
Classification of normal and skin cancer tissues using an SVM classifier. The APSD values obtained at three wave bands are used as the feature parameters for the SVM input. An optimal hyperplane constructed in the corresponding feature space determines the output.

Support vector machine (SVM) analysis is one of the simplest machine learning classification method that is supported by rigorous mathematical theory, is highly interpretable, and can identify the key factors for classification tasks. In this study, a SVM classifier was applied to perform automatic different types of tumors classification by combining relative APSD values at different wavelengths. The APSD values obtained in the three wave bands (1200 to 1240 nm, 1600 to 1700 nm, and 1300 to 1340 nm) were used as the input characteristic parameters for the classification of different types of skin tissues, thus realizing their automatic discrimination, as shown in Fig. 3. The input data comprised 39 datasets with 21 features. Considering the limited number of clinical skin tissue samples available for categorization, we used the C-type SVM model with a radial basis function (RBF) kernel, which is suitable for classifying small amounts of data. The RBF kernel can be defined as follows:

$$K(x_{i},x_{j})=\exp(-\gamma {\left\|x_{i}-x_{j}\right\|}^{2}),\;\gamma >0,$$ (2)

where x is the variable matrix, i and j are the indices of the matrix, and γ is the kernel parameter. There are two parameters for an RBF kernel: the regularization parameter C and kernel parameter γ. Parameter selection for optimal SVM categorization was achieved by grid searching^59^ using cross-validation. Various pairs of (C,γ) values were tested, and the pair with the best cross-validation accuracy was selected. To increase the classification reliability, a three-fold cross-validation approach was applied by rotating the datasets used to train and test the SVM model. The samples were randomly divided into two groups: two-thirds of the samples were used for training, and the remaining samples were used for prediction. This process was implemented thrice, with each group being tested once. Finally, the performance of the classifier model was evaluated using confusion matrices and receiver operating characteristics (ROCs) curves.

### 2.6 Histopathology

**Fig. 4:**
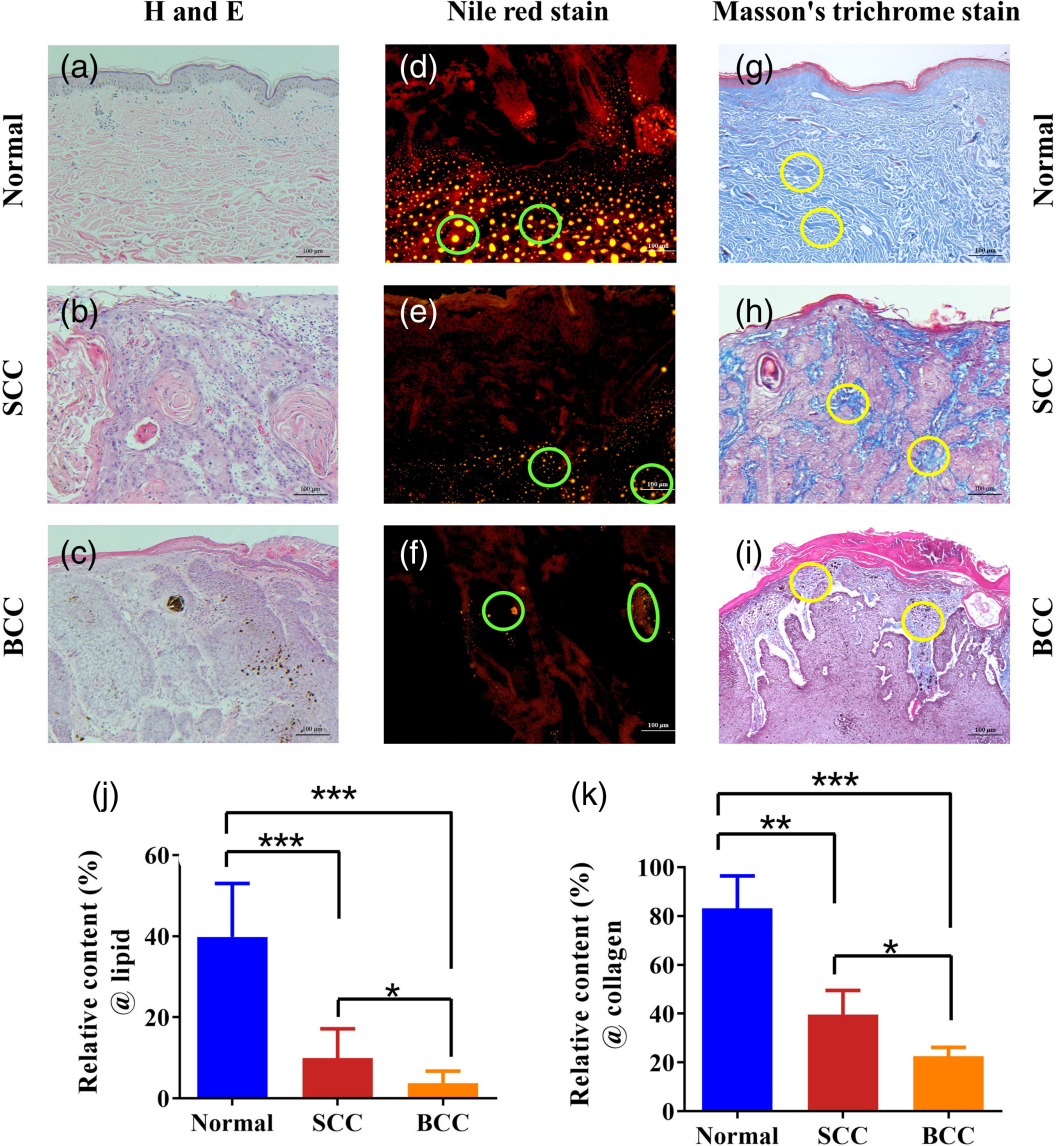
Histological results. (a)–(c) Histological examination of normal, SCC, and BCC tissues using H&E staining. (d)–(f) Histological examination of the lipid contents in normal, SCC, and BCC tissues using Nile red staining. (g)–(i) Histological examination of the collagen contents in normal, SCC, and BCC tissues using Masson’s trichrome staining. The green and yellow circle indicate the lipid and collagen clusters, respectively. (j) and (k) Statistical results of the positive-stained areas for the relative lipid and collagen contents of skin tissues obtained by histological images (*p<0.05, **p<0.01, ***p<0.001, t-test was conducted using GraphPad Prism 9.0).

To validate the PA measurements, the samples were collected and prepared for histological analysis after performing the PA measurements. The pathological analysis was divided into two parts. Some tissue samples were fixed by immersion in a 10% formalin solution and subsequent sectioning after tissue dehydration and paraffin embedding, completing the production of paraffin sections. Next, the sections were stained with hematoxylin and eosin (H&E) and others stained with Masson’s trichrome. Masson’s trichrome can stain collagen with blue color, which can be used for collagen detection. The other tissue sample was treated with an optical coherence tomography embedding agent (optical cutting temperature compound, Sakura Americas, New Mexico, United States) and flushed with distilled water to obtain frozen sections. Serial longitudinal sections (5  μm thick) were subsequently cut and stained with Nile red for lipid detection. Nile red can stain the lipid with yellow color. The staining results were observed by microscopy, as shown in Fig. 4. The relative lipid and collagen contents of the skin tissues were obtained from histological images. The ratio of the positively stained area to the whole area in the histological image, calculated using ImageJ software for each sample slice, was used as the gold standard for the relative content of collagen or lipid in the skin tissues, presenting a quantitative comparison with the relative APSD results.

## 3 Results

### 3.1 PAPCSs of Different Types of Skin Tissue

**Fig. 5:**
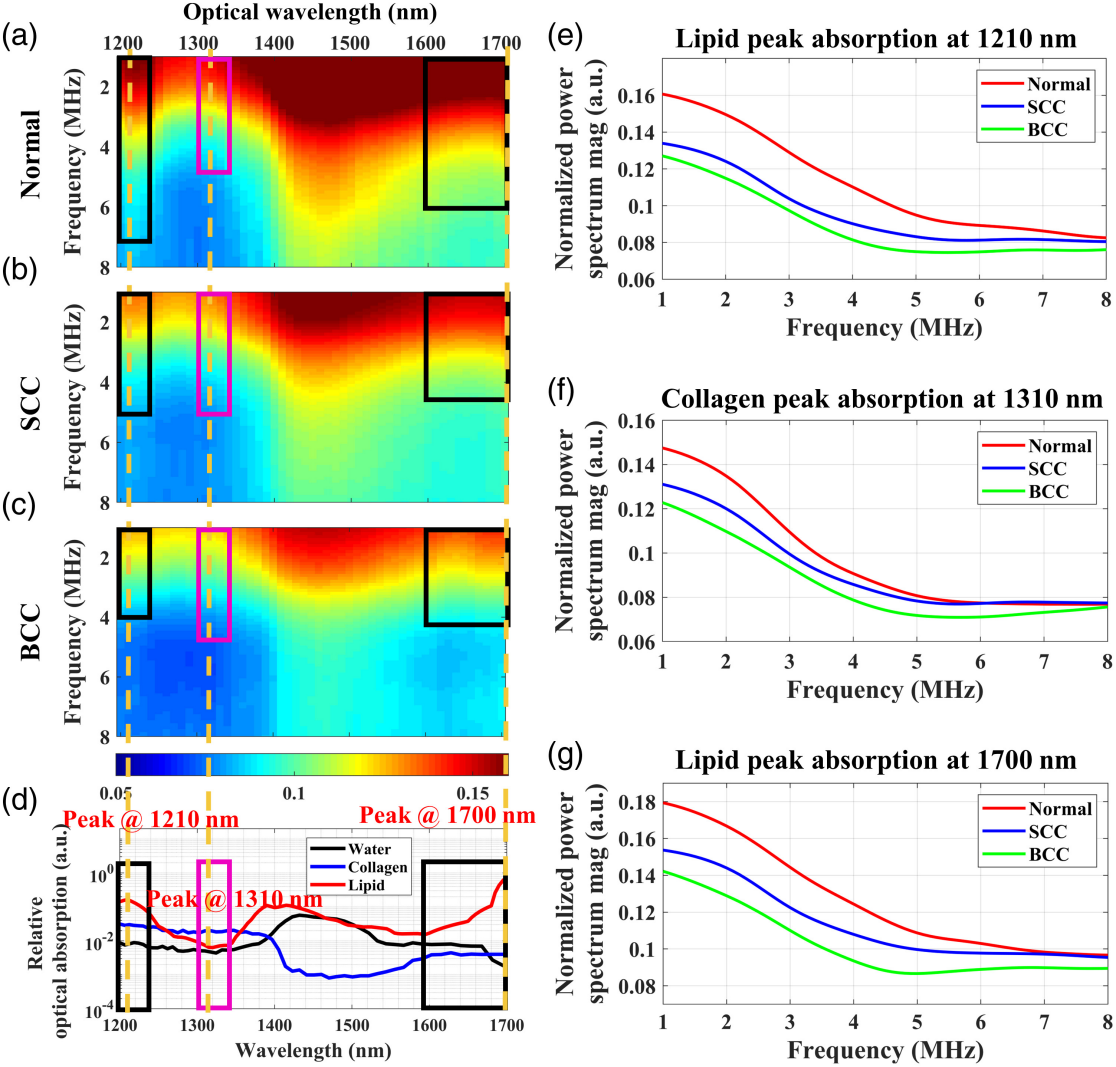
Averaged PAPCSs of different skin tissues: (a) normal skin tissue, (b) SCC, and (c) BCC. (d) Optical absorption spectra of lipid, collagen, and water in the 1200 to 1700 nm wavelength range.^25^^,^^60^ The regions indicated by black boxes (1200 to 1240 nm and 1600 to 1700 nm) correspond to regions with strong lipid absorption. The regions indicated by magenta boxes (1300 to 1340 nm) correspond to strong collagen absorption. The yellow dashed lines indicate the peak lipid or collagen absorption within each region. (e)–(g) Averaged PA power spectrum of different skin tissues at the absorption peaks of lipid (1210 and 1700 nm) and collagen (1310 nm).

Figures 5(a)–5(c) show the averaged PAPCSs of the normal, SCC, and BCC tissues, respectively. Compared with the normal tissue, in the 1200 to 1240 nm and 1600 to 1700 nm wave bands, the PAPCSs of tumor (SCC and BCC) tissues both show lower spectral magnitudes, indicating decreases in lipid content. In addition to the changes in lipid content, the PAPCSs of tumors exhibit differences in the collagen absorption wavelength range (1300 to 1340 nm). The PAPCSs of SCC and BCC show lower PA spectral magnitudes, indicating reductions in collagen contents. Interestingly, compared with the PAPCS of the BCC tissues, that of the SCC tissues shows higher spectral magnitudes in the lipid- and collagen-absorption wavelength ranges, implying that SCC tissues contain higher lipid and collagen content than BCC tissues. Specifically, Figs. 5(e)–5(g) show the PA power spectrum of different skin tissues at the absorption peaks of lipid (1210 and 1700 nm) and collagen (1310 nm), respectively. The pathological images of the skin tissues [Figs. 4(d)–4(i)] verify these differences in the lipid and collagen contents of the three types of skin tissues. The differences in PAPCSs between different skin tumors suggest that lipid and collagen in TMEs can be used as biomarkers of tumor diversity.

### 3.2 PASA Semi-Quantitative Results

**Fig. 6:**
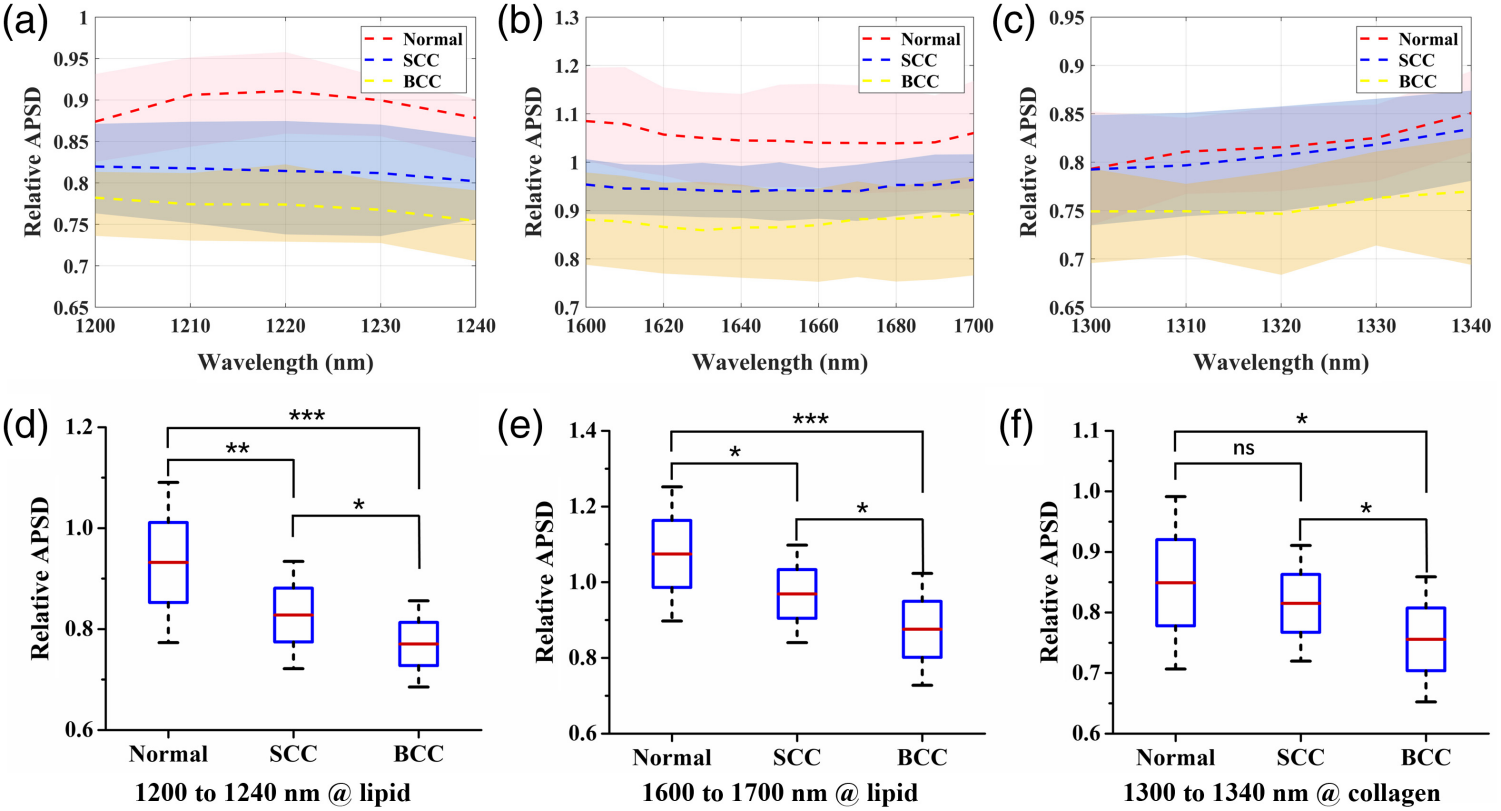
(a)–(c) PA-related APSD values with wavelengths in different skin tissues. The dashed lines indicate the averaged PA spectra, and the shaded areas overlapping the lines indicate the standard deviations. (d)–(f) Statistical results of the PA-related APSD values for the lipid and collagen contents in different skin tissues (*p<0.05, **p<0.01, ***p<0.001, ns: no statistical significance).

Based on the PASA results for skin tissues obtained at different wavelengths, the relative APSD was calculated to semi-quantify the relative lipid and collagen contents in the skin tissues. We calculated the wavelength dependence of the relative APSD for each skin tissue sample. Figures 6(a)–6(c) show the content characterization fluctuations at different wavelength. Thus, we averaged the relative APSD values in the absorption band of lipid or collagen to eliminate the measurement error caused by a single wavelength. Figures 6(d)–6(f) show the statistical results for the averaged relative APSD values, which can be used to distinguish between different types of skin tissues. As shown in Figs. 6(d) and 6(e), the lower lipid content of tumors leads to a decrease in its spectral amplitude and, consequently, a lower relative APSD for the 1200 to 1240 nm and 1600 to 1700 nm wavebands. The relative APSD values of the tumors are also lower for the 1300 to 1340 nm wavelength band, corresponding to their decreased collagen content, as shown in Fig. 6(f). In addition, different types of tumors exhibit significant differences in their collagen and lipid contents (p<0.05). For example, as shown in Figs. 6(d)–6(f), compared to those of SCC tissues, the relative APSD values of BCC tissues are lower in the regions corresponding to the optical absorption of lipid (1200 to 1240 nm and 1600 to 1700 nm) and collagen (1300 to 1340 nm), indicating that the lipid and collagen contents in the BCC tissues are lower than those contents in SCC. Figures 4(j) and 4(k) depict the relative lipid and collagen contents of the three different skin tissues obtained from the histological images and reveal notable differences. The PA results were corroborated by the changes in the lipid and collagen contents revealed in the histological images.

### 3.3 SVM Classification Results

An SVM was employed to classify skin cancers based on the lipid and collagen contents in the skin tissues. The relative lipid and collagen contents in the TME were characterized by the relative APSD values at different wavelengths. The classification results of the three-fold cross-validation approach are presented in Table 1. As shown in Table 2, the average classification accuracies of the SVM trained based only on the parameter at a single wavelength are 69.2%, 64%, and 89%. However, combining the parameters of the three bands results in an accuracy of 92.3%, the results show that the combination of multiple biomarkers can achieve more accurate intelligent diagnoses.

**Table 1:** SVM categorization. A three-fold cross-validation approach was conducted in which the samples were divided into three groups at random. Each cycle used two groups for training and one group for testing. Cate. = Categorization. Samp. = Sample.

Test sample condition	Cate. #	Test Samp. #	Cycle1				Cycle2				Cycle3			
			1210 nm	1310 nm	1700 nm	All	1210 nm	1310 nm	1700 nm	All	1210 nm	1310 nm	1700 nm	All
Normal tissue	1	1	2	3	3	1	1	1	1	1	3	2	3	3
	1	2	1	1	1	1	1	1	1	1	1	2	1	1
	1	3	1	1	1	1	1	1	1	1	1	1	1	1
	1	4	3	2	1	1	1	2	1	1	1	1	1	1
SCC	2	5	2	1	2	2	2	1	2	2	2	2	2	2
	2	6	2	3	2	2	2	2	2	2	2	2	2	2
	2	7	3	2	2	2	2	2	2	2	3	1	3	2
	2	8	2	2	2	2	3	1	2	3	2	2	2	2
	2	9	2	2	2	2	1	2	2	2	2	2	2	2
BCC	3	10	1	2	3	3	3	1	2	1	2	3	3	3
	3	11	3	3	3	3	3	3	3	3	3	3	1	3
	3	12	3	3	3	3	3	2	3	3	3	3	3	3
	3	13	2	3	3	3	1	2	3	3	3	3	3	3
Training accuracy (%)			95.8	75.8	95.8	100	92.5	100	96.7	100	83.3	96.7	92.5	95.8
Testing accuracy (%)			61.5	61.5	92.3	100	76.9	53.8	92.3	84.6	69.2	76.9	76.9	92.3

**Table 2:** Mean SVM categorization accuracy in Table 1.

	1210 nm	1310 nm	1700 nm	All
Mean training accuracy (%)	90.5	90.8	95	98.6
Mean testing accuracy (%)	69.2	64	87.2	92.3

**Fig. 7:**
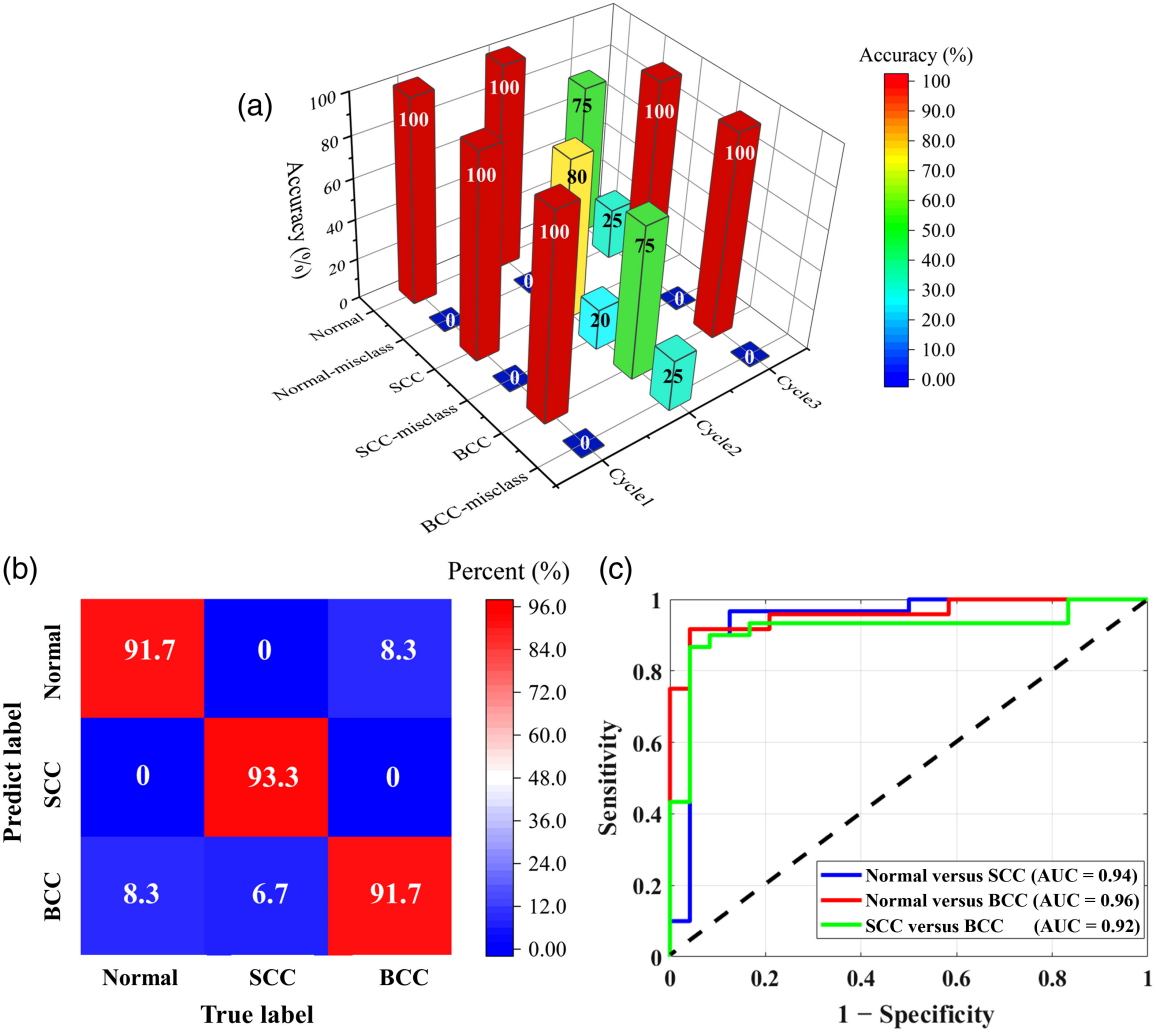
SVM classification results. (a) Classification results for normal, SCC, and BCC tissues at each fold, including the accurate classification and misclassification rates. (b) Confusion matrix for the normal, SCC, and BCC tissues. (c) Comparison of the ROC curves for the normal, SCC, and BCC tissues. AUC represents an area under ROC curve. Normal-misclass, normal-misclassification; SCC-misclass, SCC-misclassification; BCC-misclass, BCC-misclassification.

The classification performance of the SVM was evaluated using a confusion matrix, as shown in Fig. 7(b). The confusion matrix provides the classification accuracy of the SVM for individual classes. Each column of the matrix corresponds to a true label, and each row corresponds to a predicted label. The main diagonal shows the classification accuracy of the SVM for the individual classes. The off-diagonal values indicate the misclassification rates of the SVM for classifying the individual classes. The results show that SVM-based multiclass categorization can achieve diagnostic accuracies of 91.7% (normal), 93.3% (SCC), and 91.7% (BCC). The ROC curves yielded by the SVM for the normal, SCC, and BCC tissues are presented in Fig. 7(c), revealing areas under the ROC curves of 0.94, 0.96, and 0.92, respectively. Overall, SVM-based categorization proved effective in diagnosing tumors with a high level of accuracy.

## 4 Discussion

TME is a complex system in which collagen and lipid are the important components of TME. It has been reported that collagen and lipid can be used as biomarker to assess tumors.^61^^,^^62^ Several techniques have been available to detect lipid and collagen, but they all have limitations, including: invasiveness, time-consumption, and limited detection depths. Based on the contrasting optical absorption spectra of different endogenous chromophores in tissue, PA detection technique can provide rich content and microstructure information of the endogenous chromophores in biological tissues, allowing more accurate tumor diagnoses. In addition, PA has a greater detection depth than optical detection technology, benefiting from the fact that the PA signal originates from the absorption of laser energy by biological tissue, independent of the phase of the light wave.

Experiments on different types of skin tissues showed that the PAPCS obtained by PASA contained rich diagnostic information. The loss of collagen and lipid content can be observed in the corresponding PAPCS and can be used to characterize different types of tumors. Owing to the differences in lipid and collagen content of different tumors, each type of tumor has a unique PAPCS. The PASA parameter APSD is correlated with the collagen and lipid contents. The decreases in the lipid and collagen contents in skin cancer tissues cause the PA spectral amplitude of the power spectrum to decrease, corresponding to a lower relative APSD value. Furthermore, SCC originates from higher differentiated epithelial cells than BCC, with higher amounts of lipid and collagen,^63^ resulting in their APSD values higher than those for BCC in the absorption band of lipid and collagen. Statistical results of the content and microstructure changes of endogenous chromophores related to histology can be obtained according to PASA parameters. Thus, PASA provides an objective method of classifying SCC and BCC independently of the experience of a physician or pathologist. In addition, the PASA eliminates the effects of noise and system errors, providing system-independent semi-quantitative results. Our results suggest that PASA can provide sensitive, semi-quantitative content of endogenous chromophore in tissues to achieve non-invasive diagnosis and identification of different types of tumors.

Frequency spectrum of PA signal is related to the size and content of the optical absorber. In addition, our previous work has investigated the frequency anisotropy caused by the direction of the structure^35^. There appeared well distributed and more parallel collagen in normal skin tissues. While in the tumor tissue (SCC and BCC), the collagen was obviously unorderly and the amount of collagen appears reduced.^64^ There was no statistical difference in the structure and distribution of collagen cluster between SCC and BCC. In the future, we can use multiple parameters to characterize the distribution and content of collagen and lipid simultaneously to better identify tumor tissues from normal tissues.

Machine learning is an effective feature extraction tool. Even in small samples, the characteristics of PA signals can be fully learned to achieve good disease diagnosis, and machine learning has been widely used in the detection of various diseases.^65^^,^^66^ In this study, an SVM, one of the simplest machine learning tools, was utilized to distinguish automatically between different types of tumors based on the extracted PA parameters. The proposed method takes advantage of quantitative parameters of multiple wavelengths, contains rich diagnostic information, and improves the separation of high-dimensional datasets. As the number of clinical skin tissue samples was limited, we used the SVM algorithm with the RBF kernel, which is appropriate when the amount of data to be classified is small. Furthermore, to prevent overfitting based on a small number of training datasets, K-fold cross-validation was performed. The choice of the number of K-fold depends on the sample size, number of parameters, structure of data, and so on.^67^ Due to the limitation of the sample size, three-fold cross-validation was used in our study. In future work, more training datasets should be included and a higher and appropriate K-fold cross-validation should be selected, which would improve the robustness of the SVM model and make the classification of skin cancer types using the SVM approach more accurate.

Considering the accessibility of human tumor samples, the object of this study is human skin tumor tissue *in vitro* for pathological examination. *In vitro* experiments significantly improved the operability of the samples and the stability of the experiments. However, the disadvantage is that the optical absorption of hemoglobin cannot be measured. The optical absorption of hemoglobin is dominant in the visible to near infrared wavelength range (i.e., 400 to 900 nm). Therefore, the results acquired at the wavelengths of 1200 to 1700 nm were less affected by the hemoglobin. In the future, we plan to use the PASA method *in vivo*. Considering the influence of water *in vivo* experiment, which has high absorption in the 1200 to 1700 nm, a spectral unmixing procedure should be used to enable more precise evaluation of the relative contents of lipid and collagen. In fact, this approach also has considerable potential to be extended to the diagnosis of other types of cancers with altered collagen or lipid contents, e.g., breast cancer,^12^ prostate cancer.^13^^,^^14^. Furthermore, there were clear differences in the PA power spectrum between skin cancers and normal tissues. This finding not only confirms that the PA signals of tumor and healthy tissue are different but also suggests the possibility of using the PASA to define tumor boundaries in the future. Currently, the primary treatment for skin cancers is surgical excision. However, the existing clinical techniques cannot accurately define the boundaries of tumors, leading to a postoperative residual tumor tissue, which may cause disease recurrence. In future studies, we also intend to apply the PASA *in vivo* to identify the boundaries of skin cancers more accurately and objectively, to help doctors remove such tumors completely and reduce complications.

## 5 Conclusion

In summary, we introduced the PASA method to characterize the content of endogenous chromophore in the TME to better understand the interaction and regulation of tumors and TME. The parameters extracted from PASA were used to semi-quantify the content of endogenous chromophore. It was found that the collagen and lipid content can be used as biomarkers of tumor diversity, and we used this to successfully diagnose different types of tumors with improved accuracy. Considering the PA technique is non-invasive and has greater detection depth than pure optical technology, the proposed method shows considerable potential for non-invasive and more accurate diagnosis of tumors *in vivo*.

## Code, Data, and Materials Availability

The data that support the findings of this study are available from the corresponding author upon reasonable request.
